# FAANG, establishing metadata standards, validation and best practices for the farmed and companion animal community

**DOI:** 10.1111/age.12736

**Published:** 2018-10-12

**Authors:** P. W. Harrison, J. Fan, D. Richardson, L. Clarke, D. Zerbino, G. Cochrane, A. L. Archibald, C. J. Schmidt, P. Flicek

**Affiliations:** ^1^ European Molecular Biology Laboratory European Bioinformatics Institute Wellcome Genome Campus, Hinxton Cambridge CB10 1SD UK; ^2^ The Roslin Institute and Royal (Dick) School of Veterinary Studies University of Edinburgh Easter Bush Midlothian EH25 9RG UK; ^3^ Department of Animal and Food Sciences College of Agriculture and Natural Resources University of Delaware Newark DE 19716 USA

**Keywords:** community standards, FAIR principles, farmed animals, functional annotation, genome to phenome, livestock, metadata validation

## Abstract

The Functional Annotation of ANimal Genomes (FAANG) project aims, through a coordinated international effort, to provide high quality functional annotation of animal genomes with an initial focus on farmed and companion animals. A key goal of the initiative is to ensure high quality and rich supporting metadata to describe the project's animals, specimens, cell cultures and experimental assays. By defining rich sample and experimental metadata standards and promoting best practices in data descriptions, deposition and openness, FAANG champions higher quality and reusability of published datasets. FAANG has established a Data Coordination Centre, which sits at the heart of the Metadata and Data Sharing Committee. It continues to evolve the metadata standards, support submissions and, crucially, create powerful and accessible tools to support deposition and validation of metadata. FAANG conforms to the findable, accessible, interoperable, and reusable (FAIR) data principles, with high quality, open access and functionally interlinked data. In addition to data generated by FAANG members and specific FAANG projects, existing datasets that meet the main—or more permissive legacy—standards are incorporated into a central, focused, functional data resource portal for the entire farmed and companion animal community. Through clear and effective metadata standards, validation and conversion software, combined with promotion of best practices in metadata implementation, FAANG aims to maximise effectiveness and inter‐comparability of assay data. This supports the community to create a rich genome‐to‐phenotype resource and promotes continuing improvements in animal data standards as a whole.

## Introduction

The Functional Annotation of ANimal Genomes (FAANG) project is a global initiative aiming to accelerate research in genome biology by creating a rich genome‐to‐phenome resource with a particular focus on farmed and companion animals (Andersson *et al*. 2015; Tuggle *et al*. 2016). A key part of the generation of this central resource is to ensure that metadata are high quality, well described, standardised and open. Although initially focused on the farmed and companion animals, these standards would generally be applicable to any vertebrate study aiming for a high level of reproducibility. It is hoped that these standards can be adopted as a benchmark for any dataset heading for an open archive. The standards are openly licensed on GitHub (www.github.com/FAANG/faang-metadata), and checklist versions that allow selection by any depositor are being made available at the European Molecular Biology Laboratory–European Bioinformatics Institute (EMBL‐EBI) archive submission services. This paper outlines the standards adopted by FAANG, the importance of validation of datasets to the rulesets and the best practices outlined for its consortium and the vertebrate genomics community as a whole. The aim of the FAANG Data Coordination Centre (DCC) is to address the challenges faced by the community in developing infrastructure capable of collectively and effectively coordinating research efforts in genome‐to‐phenotype research. The key challenges are: (i) to develop sample and experiment metadata standards to enrich data recording, (ii) to standardise global terminologies through use of ontologies, (iii) to utilise the extensive livestock datasets generated outside of the project through use of less stringent legacy standards, (iv) to ensure records are FAIR (findable, accessible, interoperable and reusable), (v) to develop validation software to support the community in meeting the metadata standards and to submit their data to the public archives, (vi) to provide active support to the community and (vii) to provide a community data portal that collates all sample and experimental datasets through a single focused interface.

## The sample and experiment metadata standards

The first version of the FAANG metadata standards was released in early 2016 following extensive discussions and community engagement by the FAANG Metadata and Data Sharing Committee, one of the four working groups of FAANG (see Appendix S1 for a full list of members as of February 2018). The starting point for the standards developed by the working group were those developed as part of the EU‐funded BLUEPRINT project, a component project of the International Human Epigenome Consortium, which focused on reference epigenomes from cell types and diseases of the haematopoietic system (Martens & Stunnenberg 2013; Fernández *et al*. 2016). This set the initial precedents and basis for discussion but obviously required significant refactoring and extension to be applicable to samples from the diverse species that comprise farmed and companion animals and the broad functional annotation aims of FAANG.

The FAANG standards were designed to set a high bar for members of the consortium, with a focus on ensuring rich data descriptions that would enable powerful cross‐depositor analyses to be performed. The standards continue to evolve, under the guidance of the FAANG Metadata and Data Sharing Committee, to reflect the non‐static nature of the field. They continually refine an appropriate level of mandatory information to both ensure data richness and maintain compliance with the provision of data from the community. The standards are version controlled in GitHub (www.github.com/FAANG/faang-metadata), with versions numbered and documented releases of the metadata specifications. At the time of writing the standards were at version 3.5, with recent releases focussing on establishing experimental metadata standards for reduced‐representation bisulphite sequencing (Meissner *et al*. 2005), whole‐genome bisulphite sequencing (Cokus *et al*. 2008; Lister *et al*. 2009; Laurent *et al*. 2010) and whole‐genome sequencing experiments. These changes reflect the emergence of these techniques as key experimental tools within the FAANG community for genome‐to‐phenome studies. The standards will continue to evolve as new technologies and experiment strategies are developed and employed by researchers. A tabular representation of the latest version of the standards is always available from the FAANG validation tools website (www.ebi.ac.uk/vg/faang/rule_sets/). This provides a more user‐friendly version of the metadata standards and is the main reference point for most FAANG consortium members. The web tabular representation is automatically generated from the underlying master JavaScript Object Notation (JSON) rule‐set documents held in the GitHub repository, ensuring that both versions remain completely in sync. The validation software developed to aid depositors also utilises the same JSON metadata standard documents held in the GitHub repository, ensuring that all depositions are always meeting the latest agreed standard.

Samples and experiments are labelled as having met the FAANG standard in the FAANG data portal and are distinguished from data that meet less stringent legacy standards (www.data.faang.org/organism). For clarity it is important to record the version of the metadata standard under which a particular sample or experiment was archived, as it is conceivable that an older sample might no longer meet the updated metadata standard. If this occurs, then the last version that the record did successfully meet will be clearly labelled in the data portal to enable users to identify the appropriate data quality. Thus far no metadata updates have invalidated existing records. It is unlikely that future updates will cause issues, as the majority of changes involve expanding the metadata specifications to include new sequencing technologies and techniques.

## Determining what is required

During the process of establishing a metadata standard, one of the most important considerations is determining which fields are essential for both the project's success and ensuring future reusability of the data. Setting an appropriate bar for mandatory information from submitters is crucial for ensuring data richness while not making requirements so time consuming or cumbersome that they deter scientists from engaging with the project and providing data. For FAANG to be successful in enabling cross‐depositor analysis, a comprehensive metadata standard has been set. To help meet the standard and to minimise the time necessary to complete the requirements, the FAANG DCC has provided metadata validation and conversion tools (www.ebi.ac.uk/vg/faang). These accept completed spreadsheet templates or data provided programmatically in JSON format. This tool validates and then converts the provided metadata to the formats required for submission to the appropriate EMBL‐EBI archive. This software will be described in more detail later.

In addition to the standard mandatory and optional field distinctions, FAANG has implemented an additional intermediate field status of ‘recommended’. Recommended fields are those deemed important for downstream analyses and reproducibility but are not possible for every depositor to supply in every instance. For example, animal birth date is an important field for some downstream analyses, and it is expected that most submissions will include this information, but it became clear that not every submitter's records routinely included it. The recommended field category has therefore been of particular importance in these cases, where it was concluded that it was not always possible to complete fields initially flagged as mandatory. Rather than excluding these otherwise acceptable samples and experiments from the project, the fields were changed from ‘mandatory’ to ‘recommended’ by consensus of the FAANG Metadata and Data Sharing Committee. The recommended status in the metadata standards highlights the importance of the field without preventing depositors who are unable to obtain it from contributing to the project.

The metadata validation system, which will be discussed in greater detail later, warns depositors if they have not provided data for recommended fields to highlight the fields important to the project and encourage users to supply the information if it is available. Of course, fields key for reproducibility and usability issues should always remain mandatory. The distribution of mandatory, recommended and optional fields at the time of publication, with examples of each type of field in parentheses, is shown in Table 1.

**Table 1:** The distribution of mandatory, recommended and optional fields at the time of publication. In parentheses are examples of the fields in each category

Field category	Animals and samples (*n*)	Experimental assays (*n*)
Mandatory	27 (sex, organism, breed, developmental stage)	35 (assay type, extraction protocol, experiment target)
Recommended	7 (birth date, health status, publication)	14 (sequencing date, sequencing location, restriction enzyme)
Optional	32 (birth location, specimen volume)	3 (sample storage, library selection)

It will not be possible for every submitter to complete every field, but not providing data can occur for a variety of reasons. It is important to downstream data consumers to understand if the data were really missing, withheld or potentially obtainable in a future update or on request. FAANG has established a defined set of controlled terms to describe missing data, making the standardised reasons clearly understandable and searchable across the resulting datasets. The four defined terms are: ‘not applicable’, ‘not collected’, ‘not provided’ and ‘restricted access’. ‘Not applicable’ is employed when the field does not apply to this sample. ‘Not collected’ implies that there is no expectation that the field can ever be provided. ‘Not provided’ describes the possibility that the field may be added later or at least be obtainable by contacting the original submitter (this is possible because all submissions also include contact information). ‘Restricted access’ refers to fields where the value exists but it cannot be included in a public document. These are the only missing data terms accepted by the FAANG metadata validation system and, importantly, the four fields interact differently with it. For example, for recommended metadata fields, supplying the value ‘restricted access’ or ‘not applicable’ will pass the metadata standards but a value of ‘not collected’ or ‘not provided’ would display a visual warning to the submitter to supply the missing information if possible. This system encourages, when appropriate, depositors to supply the additional information for recommended fields. As these are not mandatory fields, the submission of the sample or experiment can still proceed by entering one of the four possible missing data terms.

The FAANG submission tool set, and the archives to which it submits, do not limit users to supplying only data that are in the metadata standards; both also allow any additional fields not covered by the standard to be submitted. This includes the ability to submit these additional fields using supplied ontologies to standardise the provided metadata and benefit from links to the ontology database descriptions and resources. The FAANG Metadata and Data Sharing Committee and DCC regularly review these additional fields supplied by depositors to spot instances when the fields should be incorporated into the metadata standards and values more tightly controlled.

During sample and experiment registration, individual, institutional and sequencing centre details are recorded for each entry to help ensure data provenance. All individuals associated with a sample or experiment also have their organisational role, such as submitter, data analyst or experiment performer, recorded using the ontology terms in the Experimental Factor Ontology (child terms of www.ebi.ac.uk/efo/EFO_0002012). This makes it clear who contributed to the work and, for downstream consumers, who to contact for further information or collaboration.

## Importance of ontologies

The truly international nature of the FAANG project requires data standards that cope with country‐ and language‐specific terminology and breed definitions. This is in addition to the standard scientific complexities of accurately recording a suite of diverse sample and experimental characteristics. To this end, FAANG extensively employs ontologies to converge on a common set of terminologies for all its descriptive fields. The power of ontologies for enhancing and standardising data coordination has been widely documented (Ashburner *et al*. 2000; Cote *et al*. 2006; Smith *et al*. 2007; Malone *et al*. 2010).

Users are encouraged to research and identify the most specific ontology terms for their submissions using search tools such as the Ontology Lookup Service (Jupp *et al*. 2015). The FAANG DCC is focussing on continually improving both the experience of its depositors and the quality of the provided metadata. FAANG depositors, with the support of the Ontology Lookup Service, are researching and selecting high quality ontologies, but we recognise that provision of these data can be time consuming. We are therefore currently looking to incorporate the EMBL‐EBI tool zooma (www.ebi.ac.uk/spot/zooma/) into the FAANG validation software. This service maps free text annotations from depositors to the most optimal ontology terms based on a curated repository of annotation knowledge. zooma automates ontology selection, continually improves its predictive ability based on previous selections and greatly improves the accuracy and specificity of the submitted metadata.

A key part of the process is that the FAANG validation tools are ontology aware and have metadata rules based on ontology hierarchies. For example, for values for ‘health status at collection’ the metadata standard requires any supplied value to be a descendant of the ontology terms ‘http://purl.obolibrary.org/obo/PATO_0000461’ (normal) or ‘http://www.ebi.ac.uk/efo/EFO_0000408’ (disease). Any term that is a ‘child’ of these terms in the hierarchy is accepted. Ontology databases for disease‐focused model organisms, such as human and mouse, are far more complete than for farmed and companion animals, some of which are not mammals, and have more in‐depth associated descriptions and synonyms. Through its extensive requirement for ontologies, FAANG members are regularly contributing improvements to the ontology databases FAANG supports. This enhances the quality of farm‐ and companion‐animal‐specific terminology within the ontologies. For example, FAANG members were the first to add buffalo breeds to the Livestock Breed Ontology, and their initial four registered breeds (Mediterranean, Pandharpuri, Jafarabadi and Bhadawari) have now increased to 11. Together these updates will improve the future provision of standardised metadata ontology descriptions.

## The complexity of breeds

One of the most challenging fields for standardisation of farmed and companion animal data from across the globe is the organism's breed. Breeds are often defined at a national level by Food and Agriculture and other bodies, including breed societies, which have local variations and common shared names between species. Breeds often therefore do not fully account for genetic similarity and yet remain incredibly important for identity data discovery and classification. Breeds also often incorporate regional subdivisions, with breeds located or arising from a particular geographic region, which can then have strong branding and economic importance that is protected at a national and international level. Further complexities are added by considering distinctions between the numerous populations across different countries formed by different breed‐crossing strategies and also any original indigenous populations. It is therefore a challenging and sensitive area for global standardisation but important that identification and commonality can be established for the breadth of FAANG samples.

Full adoption of a single standard breed name is unlikely to be possible and is therefore outside of the scope of FAANG's standardisation efforts. FAANG is however committed to annotating and hierarchically defining as clearly as possible the breadth and diversity of the breeds represented in FAANG studies and datasets. Although ontologies allow the addition of synonyms so that country and regional specific variations in a breed can be captured, the requirement for a primary name could potentially cause political issues for which name has precedence. Our approach is to allow depositors the option to submit their own preferred name for every ontology field. We can then display their breed name of preference whilst retaining the ontology ID to allow programmatic linking to breeds under the same or related terms through the ontology hierarchy. Although not a perfectly unified solution, it is an important progression over non‐hierarchical free text breed fields, and employing it provides significant benefit. The Food and Agriculture Organisation of the United Nations (FAO) has launched a number of initiatives to support and protect the world's animal genetic resources (FAO 2007). A key part of this is to understand the breed diversity across Europe, the importance of local breed distinctions and support for rare or endangered breeds. Whilst pursuing and supporting the establishment of the Livestock Breed Ontology, FAANG will also liaise, cooperate and support the efforts of the FAO as well as European and national bodies to make progress on this significant challenge in quantifying breed diversity through the establishment of a common ontological language.

An additional complication in the standardisation of breeds in farmed animal research is the recording of cross‐breeds. Cross‐breeds are the offspring resulting from two different parental breed lineages, often but not always purebred, and are a particular challenge for metadata recording and subsequent data filtering. FAANG has developed a nomenclature for cross‐breeds within its sample rules to standardise the way that these individuals are recorded. All cross‐bred animals are annotated with the livestock breed ontology (LBO) term ID for ‘crossbreed’ specific to their species, for example, LBO_0001036 for cattle crossbreed and LBO_0001038 for goat crossbreed. When the breeds of the parent animals are known, the progeny of two purebred animals are described with a custom breed name using the format ‘breed sire × breed dam’. In this format the sire is always listed first; for example, this could be ‘Texel sire × Scottish Blackface dam’. Further instructions are provided for cases when the breed of one of the parents is not known or if the parents were themselves cross‐breeds. Full guidance for recording cross‐bred animals is available on the FAANG wiki pages (https://www.ebi.ac.uk/seqdb/confluence/display/FAANG/FAANG+guidelines+for+livestock+breed+nomenclature).

## Legacy standards

The FAANG standards are designed to encompass all forms of livestock sampling and experimental data including anything produced over the preceding decades prior to FAANG's formation. This historical data are not expected to meet the strict FAANG metadata standards applied to modern sampling and experimental datasets. FAANG has therefore additionally included legacy standards (https://github.com/FAANG/faang-metadata/tree/master/rulesets), which through careful consideration by the Metadata and Data Sharing committee focuses on a minimal number of required fields, with the remainder as optional. This has the important distinction that not all data are suitable to be included in the project. However, these datasets still have to meet this reduced minimal standard that means the data is useful for analyses. The usefulness of legacy data will depend largely on the analysis in question, and it is therefore important that data are clearly marked on the portal as to what standard they meet with easy filtering options to include or exclude the legacy data. The legacy rules are incorporated into the automated import scripts that query the EMBL‐EBI archives for appropriate data. This ensures that appropriate data produced outside of the FAANG consortium can be made available for comparative genome‐to‐phenome analyses.

## FAANG conforms to FAIR standards

The FAIR standards (Wilkinson *et al*. 2016) are a guiding set of principles that state that all research objects should be findable, accessible, interoperable and reusable. This supports reuse by individuals and importantly enables computational automatic identification and reuse of datasets. FAANG conforms to the FAIR gold standard in that the data are openly accessible and functionally interlinked. It achieves this through the relationship hierarchy of its accessions in that all samples and experiments are linked through unique biosamples and study identifiers. The FAANG data portal interface goes beyond open data access to a rich functional interlinking that can be explored manually and programmatically through its integrated platform. Each sample is clearly linked to the datasets produced from it, and its relationship to all other samples, experiments and family relationships can be traced. There is a strong provision for programmatic interactions with the FAANG data, with clear API (application programming interface) interactions built on an Elasticsearch query interface. Direct download links to the datasets are provided within the Elasticsearch API calls and within the summary pages on each entry on the portal (http://data.faang.org/help/api). FAANG promotes the reuse of data and encourages large‐scale cross‐depositor analyses through its rich metadata schema and clear data sharing principles (https://www.faang.org/data-share-principle). This statement is based upon the Fort Lauderdale principles, and it encourages the community to register samples as soon as possible after collection and deposit assay data in archives prior to publication for the benefit of the entire livestock community and the acceleration of research. The FAANG standards are included in the FAIRsharing initiative (https://fairsharing.org/bsg-s000672; https://fairsharing.org/bsg-s000673), a curated educational resource on inter‐related data standards, to provide a rich FAIR vertebrate standard to promote data sharing worldwide.

## Software to support depositors and validation

To obtain rich and accurate metadata, the software that supports the depositors in the provision of the metadata is as important as the quality of the standard itself. Rich metadata requirements can be challenging to meet, particularly for submitters handling large quantities of samples and experiments. Using programmatic interfaces, such as API submissions, to solve batch submissions is important, but for experimental groups lacking informatics expertise, it does not resolve this issue. A fully manual or web‐based form submission, without the bulk submission assistance that the FAANG tools provide, will likely have inaccuracies as it grows beyond tens of records. This becomes a severe issue as the number of submitted records approaches hundreds to thousands of samples, a size of submission already seen multiple times in FAANG.

For programmatic submitters, the FAANG DCC has provided a full API interface that accepts and returns validated JSON documents for ease of programmatic parsing. The API can also be used to convert the submitted template spreadsheet or JSON metadata documents into the format required for submission to the archives. However, a significant number of FAANG research submitters are wet‐lab biologists rather than bioinformaticians. Therefore, the FAANG DCC has developed a submission process that accepts microsoft‐excel‐based batch submissions. For both the sample and experimental rulesets, excel templates are provided to capture the FAANG required metadata. Drop‐down boxes within the excel templates and extensive documentation are provided to support accurate completion of the templates (https://www.ebi.ac.uk/seqdb/confluence/display/FAANG/FAANG+Archive+Submission+guidelines). The completed templates can be validated by the FAANG validation service (https://www.ebi.ac.uk/vg/faang/validate/) with an interactive web‐based results page that details the errors and provides warnings that require fixing. Alternatively, the service can provide an annotated version of the excel template detailing the errors and warnings. Once a submission fully meets the ruleset, this same template can be used in the conversion service (https://www.ebi.ac.uk/vg/faang/convert/) to be converted into the file formats required by the archives.

The strategy is to enable researchers to enrich and improve their own data, as they have the most knowledge about their samples and experiments. The aim is to bridge the gap between data producers and consumers to ensure a fair balance between their respective needs. The FAANG DCC is currently investigating ontology lookup from input‐free text utilising the EMBL‐EBI's zooma service.

## FAANG Data Coordination Centre

The FAANG DCC implements the direction and decisions determined by the FAANG Metadata and Data Sharing committee. It manages changes and versioning of the online metadata rulesets and develops the programmatic and interactive validation and conversion tooling provision of accurate and rich metadata. It is also responsible for development of the FAANG data portal for provision and access to all FAANG sample and experimental data. The direction, scope and accountability of the FAANG DCC is maintained by the Strategic Management Committee, which meets annually to review the progress of the DCC and advise on proposed developments for the following year. The DCC actively promotes the importance of rich, accurate and ontology‐supported metadata at high‐profile livestock conferences and through FAANG communication lines. The provision of a single portal through which validated and clearly labelled farmed and companion animal data can be identified, filtered and downloaded promotes the community members to continually improve their own standards. This will enhance the reproducibility and citation of high quality research and encourage users to meet the FAANG standard so that their data are included in FAANG meta analyses.

A key development for FAANG in the coming years will be the planned developments for a single unified submission interface for all EMBL‐EBI archives. This will replace the current scenario of a different submission interface for each archive. This simplifies the submissions process for the user and allows sample and experimental information destined for different EMBL‐EBI archives to be packaged as a single submission. The FAANG DCC will continue to provide a high level of support to FAANG submitters and provide access to the extensive documentation and training offered by EMBL‐EBI during the transition period. One key new feature is the integration of validation checklists into the submission system itself, currently performed by FAANG's separate metadata validation tools (http://www.ebi.ac.uk/vg/faang/validate/). This will be a stand‐alone service that is able to continue to provide the pre‐validation of submissions that FAANG users have benefited from and also streamline the submission process. The proposed changes will make it easier to provide support for users to submit high quality metadata. A significant advantage is that the checklist will be available to any submitter to EMBL‐EBI archives, not just to members of the FAANG consortium. This enables promotion of FAANG standards to the wider livestock and vertebrate communities. Information on the submissions process is kept updated on the FAANG wiki site (https://www.ebi.ac.uk/seqdb/confluence/display/FAANG/FAANG+Wiki+Home).

## The FAANG data portal

The FAANG data portal (http://data.faang.org/home) provides a focused functional data resource from which researchers can explore, identify and download validated, richly described, high quality and comparable datasets for their genome‐to‐phenome research. The centralised resource automatically identifies, classifies and validates sample and experimental data from the public data archives to import into the portal. The records pages provide users with filters to aid in the identification of datasets of interest and whether to exclude data that meet only the legacy standards. Each individual record contains direct links for downloading the analysis data files from the public archives. The search function includes an intuitive autocomplete feature that simultaneously searches across samples, experiments, files and datasets. For programmatic users, the portal includes an API that has access to search across all FAANG fields.

## Conclusion

Any researcher can sign up to take part in FAANG activities and also join the respective working groups to shape the direction and scope that the project takes. All are therefore welcome to contribute to the contents and shape the metadata standards for FAANG and the farmed and companion animal communities as a whole. The FAANG DCC and the Metadata and Data Sharing Committee are working to promote best practices in the farmed and companion animal community, rich and functionally interlinked metadata and open prepublication data sharing. The FAANG metadata standards have been adopted as the basis of the Functional Annotation of All Salmonid Genomes (FASSG) project's aligned efforts in building a rich genome‐to‐phenome resource for the salmonid community. This highlights the demand for standards beyond the FAANG community. The integration with EMBL‐EBI's new unified submission interface will address this need. The FAANG main and legacy standards, through their incorporation into the EMBL‐EBI unified submission interface, enables submitters from other domains to select to meet these rich standards and take advantage of the built‐in validation tools. In addition to specific individuals, larger communities and projects can utilise the FAANG rulesets as a basis for their own community standards. This can be achieved by them adapting and creating their own rulesets, just as the FASSG and Innovative Management of Animal Genetic Resources (http://www.imageh2020.eu/) communities have. The clear and effective metadata standards and accompanying validation and conversion tooling ensures that, from a single focal portal, the farmed and companion animal community can obtain high quality and comparable datasets for their genome‐to‐phenome research and promote continuing improvements in animal data standards as a whole.

## Supporting information


**Appendix S1.** Supplementary information.
