# Supplementary material for: FAANG, establishing metadata standards, validation and best practices for the farmed and companion animal community
Source: Anim Genet. 2018 Oct 12;49(6):520–6. doi: 10.1111/age.12736 (PMC6334167; doi:10.1111/age.12736)
Supplement: Supplementary file 1 — Appendix S1. Supplementary information. [file AGE-49-520-s001.pdf]

Appendix S1 Supplementary information

The 94 members of the FAANG Metadata and Data Sharing Committee as of February 2018 in alphabetical order. The committee is chaired by Dr Peter Harrison and Professor Carl Schmidt.

Behnam Abasht

Faisal Almathen

Andreia Amaral

Christian Anthon

Alan Archibald

Mohammad Hossein Banabazi

Konstantinos Billis

Gudrun Brockmann

Stephen Bush

Angela Canovas

Amanda Chamberlain

Kelly Chanthavixay

Emily Clark

Laura Clarke

Richard Crooijmans

Sarah Djebali

Ngoc Duy Do

Christine Elsik

Susan Fairley

Jun Fan

Marta Farre Belmonte

Adam Faulconbridge

Heather Finlayson

Carrie Finno

Paul Flicek

Elisabetta Giuffra

Jan Gorodkin

Isabelle HUE

Mathias Hansen

Peter Harrison

Xiaoxiang Hu

Zhihua Jiang

Alexander Junge

Haja Kadarmideen

Juha Kantanen

Brittney Keel

Heebal Kim

Heui-Soo Kim

Myrto Kostadima

Bharani Kumar

Danielle Lemay

Anna Lewandowska-Sabat

Sigbjorn Lien

Shikai Liu

Wansheng Liu

Ernesto Lowy

Leslie Lyons

David MacHugh

James MacLeod

Cinzia Marchitelli

Sylvain Marthey

Alisha Massa

Raluca Mateescu

Hendrik-Jan Megens

Younes Miar

Michael Mienaltowski

Chris Mungall

Kylie Munyard

Stephanie Namciu

GS Naveen kumar

Quan Nguyen

Sheila Ommeh

Alicja Pacholewska

Frank Panitz

Romi Pena

Andreas Pfenning

Kisun Pokharel

Mustapha Popoola

Hollie Putnam

Vladimir Radosavljevic

James Reecy

Kent Reed

David Richardson

Benjamin Rosen

Pablo Ross

Carl-Johan Rubin

Carl Schmidt

Steven Schroeder

Jiuzhou Song

Ian Streeter

Prash Suravajhala

Bo Thomsen

Gwenola Tosser-Klopp

Nares Trakooljul

Christopher Tuggle

Jon Olav Vik

Johanna Vilkki

Mick Watson

Klaus Wimmers

Kim Worley

Andrew Yates

Rachel Young

Daniel Zerbino

Huaijun Zhou
